# Synaptic sensitization in the anterior cingulate cortex sustains the consciousness of pain via synchronized oscillating electromagnetic waves

**DOI:** 10.3389/fnhum.2024.1462211

**Published:** 2024-09-11

**Authors:** Richard Ambron

**Affiliations:** Department of Cell Biology and Pathology, Vagelos College of Physicians and Surgeons, Columbia University, New York, NY, United States

**Keywords:** long-term potentiation, synaptic sensitization, electromagnetic waves, NMDA receptors, transcranial magnetic stimulation, consciousness

## Abstract

A recent report showed that experiencing pain requires not only activities in the brain, but also the generation of electric fields in a defined area of the anterior cingulate cortex (ACC). The present manuscript presents evidence that electromagnetic (EM) waves are also necessary. Action potentials (APs) encoding information about an injury stimulate thousands synapses on pyramidal neurons within the ACC resulting in the generation of synchronized oscillating (EM) waves and the activation of NMDA receptors. The latter induces a long-term potentiation (LTP) in the pyramidal dendrites that is necessary to experience both neuropathic and visceral pain. The LTP sensitizes transmission across the synapses that sustains the duration of the waves and the pain, EM waves containing information about the injury travel throughout the brain and studies using transcranial stimulation indicate that they can induce NMDA-mediated LTP in distant neuronal circuits. What is ultimately experienced as pain depends on the almost instantaneous integration of information from numerous neuronal centers, such as the amygdala, that are widely separated in the brain. These centers also generate EM waves and I propose that the EM waves from these centers interact to rapidly adjust the intensity of the pain to accommodate past and present circumstances. Where the waves are transformed into a consciousness of pain is unknown. One possibility is the mind which, according to contemporary theories, is where conscious experiences arise. The hypothesis can be tested directly by blocking the waves from the ACC. If correct, the waves would open new avenues of research into the relationship between the brain, consciousness, and the mind.

## Introduction

It was previously shown that the sensation of pain after an injury requires both the activation of pyramidal neurons in an area of the anterior cingulate cortex (ACC), as well as the generation of a local field potential (LFP) in the surrounding extracellular space (Ambron, 2023). LFPs can influence the activity of nearby neurons via emphatic effects (Anastassiou et al., 2011; Anastassiou and Koch, 2015) and their potential role in experiencing pain was discussed earlier (Ambron, 2023). In addition to the LFP, however, activation of the pyramidal neurons creates electromagnetic (EM) waves that can have far-reaching effects. The first sections of the present paper present evidence from multiple areas of neuroscience and physics that these waves are also essential for the expression of pain. The second sections offer two theories as to the function of the waves and their potential link to consciousness.

Pain is a complex sensation that arises from both physical (Ambron and Sinav, 2022) and psychological causes (Eisenberger, 2012; Wager et al., 2013; Papini et al., 2025). Although the suffering from psychological pain is as real as that from a lesion, relatively little is known about the neuronal networks that are responsible for psychological pain. However, there are neuronal circuits in the brain where the information from both types of pain intersects and these will be discussed where applicable. The primary focus here is on the pain from an injury or inflammation (i.e., a lesion). Pain is unique among our senses because it persists beyond the initial event to protect the injured area from additional damage: it is also adaptive because the intensity of the pain can be influenced by present and past circumstances. Chronic pain, which persists for 3 month or longer, is a major medical problem that diminishes the quality of life with both social and economic consequences (Ferraro, 2023). At present the most effective remedies for chronic pain are analgesics containing opiates, but the abuse of these drugs can result in addiction and possible death by overdosing. Consequently, there is a real need to develop alternatives to the opiates and this requires a more complete understanding of the events involved in experiencing pain. The operative term here is experience, which is the quality of pain commonly known as painfulness or suffering. Simply put, we cannot explain how a lesion actually results in suffering, and this applies to our other senses as well. For example, we know a great deal about the electrophysiological and biochemical events that occur in the visual system, yet we cannot explain how these events culminate in experiencing the color of a flower. Determining that the experiencing pain requires the generation of EM would not only yield potential new approaches to pain management, but would also provide a model to investigate how experiences arise from our other senses.

**Figure 1:**
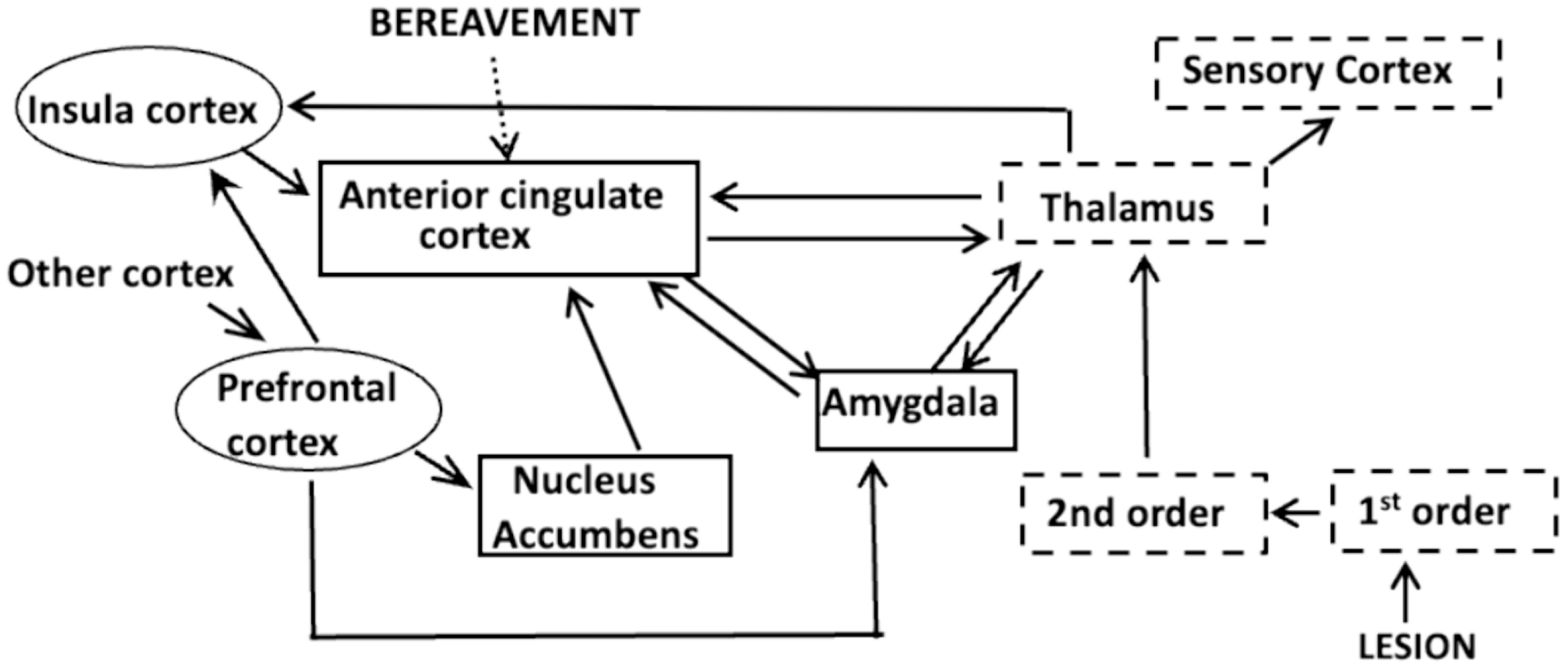
The Integrated Pain Network depicting interconnections between the components of the somato-sensory system (dashed boxes), the affective system (solid boxes) and the cognitive system (ovals). Information about an injury or inflammation is encoded in APs at the terminals of 1st order nociceptive neurons. The APs activate 2nd order neurons in the spinal chord and the information is conveyed to the thalamus which processes and disseminates the information to other cortical areas. A projection to the sensory cortex identifies the site of the lesion, whereas a connection to the amygdala can elicit fear due to memories of traumatic events. The nucleus accumbens is part of a reward network and interactions between the insula cortex and circuits in the prefrontal cortex comprise a salience network that focuses attention. Each of these circuits communicate with specific areas within the anterior cingulate cortex (acc), as do those that respond to psychological causes, such as extreme bereavement.

We know that the sensation of pain involves interactions between three neuronal systems that comprise an integrated pain network (IPN; Ambron, 2023; Figure 1). The initial event occurs in the somato-sensory system where algogenic agents released from injured tissue, or in response to an inflammation, bind to membrane receptors on the peripheral terminals of 1st order nociceptive neurons. The binding evokes action potentials (APs) that encode the severity and intensity of the lesion: the greater the number and frequency of the APs, the more severe the injury and the greater the potential intensity of the pain. The APs propagate to 2nd order neurons in the spinal cord where the initial processing of the information occurs. The resulting APs ascend to the thalamus where there is an awareness of the injury, but not its painful, or onerous aspects (Ambron, 2023). The disassociation between the awareness and the suffering is an example of sensory asymbolia (Rubbins and Friedman, 1948) and was first reported as a consequence of prefrontal lobotomies (Freeman and Watts, 1948). This means that the experience of pain arises from events distal to the thalamus.

The thalamus distributes the nociceptive information to the precentral gyrus, which alerts us to the site of the injury, and to centers in the affective and cognitive systems (Hwang et al., 2017). As first proposed by Melzack and Casey (1968) and updated by Melzack (1990) the affective system adjusts the intensity of the pain by considering the mood and circumstances in which the injury occurred. Circuits in the amygdala for example, retain a memory of traumatic events and their activation elicits fear, which exacerbates pain. In contrast, circuits in the nucleus accumbens (NucAc.) are part of a motivational system that diminishes pain if the reward is considered worthwhile (Figure 1). The insular cortex (IC) is a component of the cognitive system that receives inputs from the prefrontal cortex (PFC). Interactions between the IC and PFC are important because they decide at each instant in time which sensation will receive attention (Lu et al., 2016; Uddin et al., 2017; Figure 1). Significantly, the information from all three systems is conveyed to neuronal circuits in the anterior cingulate cortex (ACC; Figure 1).

In summary, the ultimate intensity and duration of the pain from a lesion depends on dynamic interactions between the thalamus, the spatially separated centers in the IPN, and the ACC. By applying what we have learned about these interactions, we can address three issues: First, what molecular events are responsible for the persistence of pain after a lesion? This has obvious implications for chronic pain. Second, how do the spatially and functionally distinct centers in the IPN exchange information about pain at every instant in time? And third, where does the experience, the hurtfulness of pain occur? The key to answering all three questions comes from recent advances in our understanding of the events that occur in the ACC after a lesion.

## Results

### The anterior cingulate cortex

Decades of basic research and clinical studies have established that the neuronal circuits essential for experiencing pain are located in the ACC (Shyu et al., 2010; Chapin et al., 2012; Fuchs et al., 2014; Moon and Park, 2022; Ambron, 2023). Chronic pain patients whose cingulate gyrus was surgically removed reported being aware of the lesion, but not the painfulness (Foltz and White, 1962), which is consistent with the responses from the lobotomy patients. Real time MRI studies of the placebo effect and hypnosis consistently showed increased activity in the ACC when pain is experienced, and decreased activity when the pain is attenuated (Ambron, 2023). fMRI scans also revealed that psychosocial pain increases activity within the ACC (Figure 1) (Wager et al., 2013; Xiao and Zhang, 2018) and circuits within the ACC can also modulate pain based on anxiety (Koga, 2015) and mood (Journée et al., 2023). Consequently, the ACC is where sensory, cognitive, and emotional processes come together to shape what is ultimately experienced as being painful.

**Figure 2:**
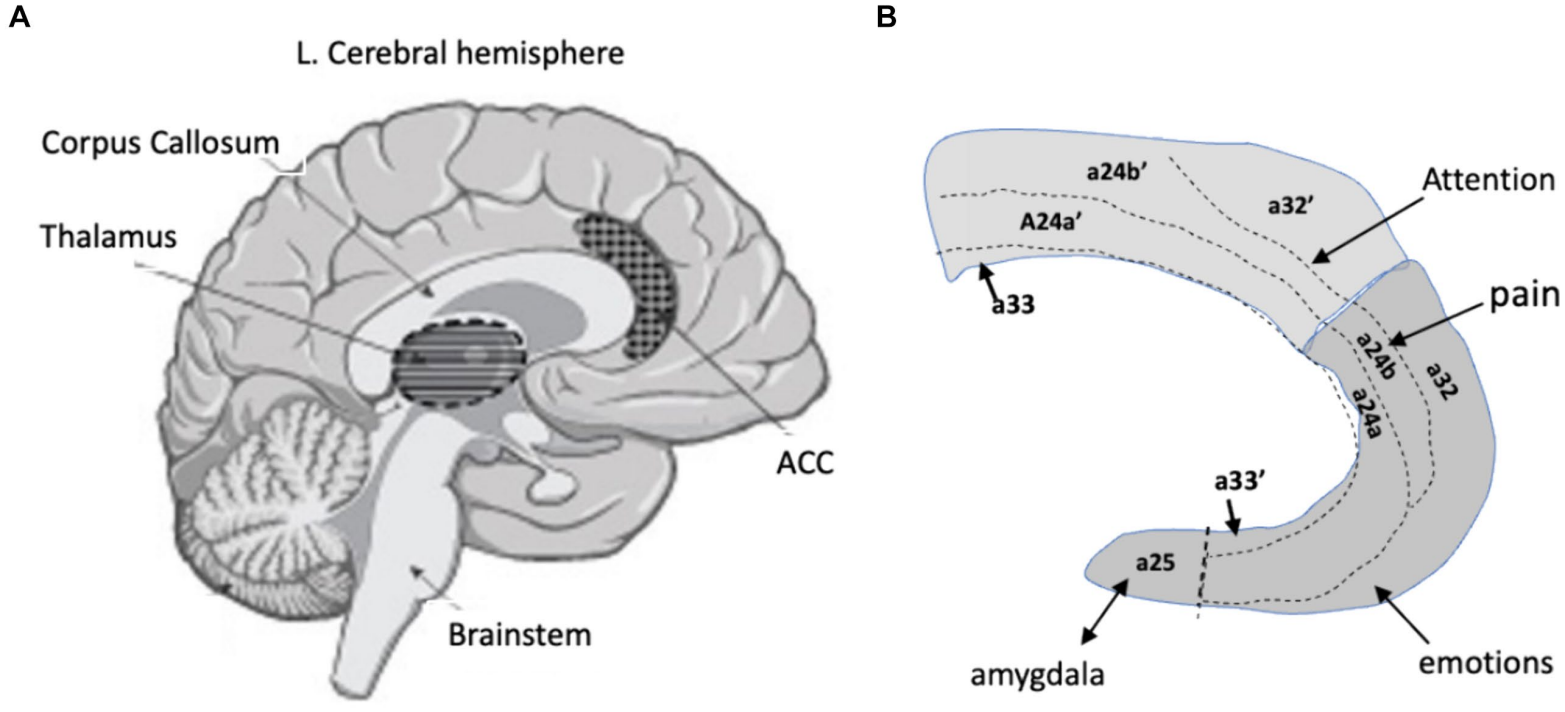
The anterior cingulate cortex. **(A)** Mid-sagittal section through the brain showing the medial surface of the left cerebral hemisphere and highlighting the thalamus and the anterior cingulate cortex (ACC), which is located above the rostral region of the corpus callosum. **(B)** The ACC showing Brodmann’s areas and the location of inputs from some of the centers in the IPN.

The ACC is heterogenous and includes primary regions 24, 25 and 32 in Brodmann’s map of the cerebral cortex (Figure 2). A major breakthrough was finding that these regions could be subdivided into discrete areas that process information about an injury or inflammation that is received from the centers in the IPN (Vogt et al., 2003; Beckmann et al., 2009; Van Heukelum et al., 2020). In particular, a center for pain in primates was assigned to the rostral area localized around 24a/b. Consequently, characterizing the function of the circuits within 24a/b should provide clues to understanding how we experience the painfulness of a lesion and might yield insights as to how these circuits mediate the psychological aspects of pain. The ACC also contains a center for attention in area 24a‘b’ that receives input from the posterior region of the IC (Lu et al., 2016; Uddin et al., 2017; Ambron, 2023). This center is near the center for pain, which is significant because a study by Fischer (2016) showed that communication between the IC and ACC is required to maintain consciousness. Notable also is that area 25, and the rostral-most regions of 24a/b, and 32 have strong reciprocal connections with the amygdala for the expression of fear (Beckmann et al., 2009). There is also an area associated with anxiety that is important due to its association with chronic pain (Koga, 2015).

The areas within the ACC are organized into local circuits consisting of pyramidal neurons, gamma-aminobutyric acid (GABA) inhibitory neurons, and interneurons that are distributed among six lamina that lie parallel the surface. The large lamina V pyramidal neurons are the primary conveyors of information throughout the cerebral cortex. Each pyramidal neuron has a single long axon that exits the circuit to communicate with distant cortical areas and a single apical dendrite that ascends to lamina I-II. Each dendrite branches extensively and intermixes with branches from adjacent pyramidal neurons creating a large receptive field (Linden et al., 2010; Pockett, 2012; Buzsáki et al., 2012; Ambron, 2023). The dendrites in lamina I-II in a24a/b form thousands of excitatory thalamo-pyramidal synapses with axons from the medial thalamic tract that transmit information about the pain from an injury. The synapses activated by APs from the thalamus contain information about an injury, but there are limited opportunities in primates to investigate the sequelae to the activation. Fortunately, area 24a/b has a counterpart, area 24b, in mice (Fillinger et al., 2017; Sellmeijer et al., 2018; van Heukelum et al., 2020) and by combining the results from studies of both primates and mice it is possible to characterize in detail the biochemical and electro-physiological processes that occur in the pyramidal neurons after an injury (Ambron, 2023). For convenience, I will designate this common area 24P.

### Long-term potentiation in the response to an injury

Activation of the thalamo-pyramidal synapses in response to an injury causes the release of the excitatory neurotransmitter glutamate (Glu) from the presynaptic terminal, thereby evoking the synchronous activation of the thousands of postsynaptic dendrites (Linden et al., 2010; Pockett, 2012; Destexhe and Bedard, 2013; Einevoll et al., 2013; Ambron, 2023). Synchronicity is important for the processing of pain information and it can be facilitated by the presence of an h-Type membrane current (Ness et al., 2018). The same oscillations and synchronicity can also be elicited experimentally by applying a Theta Burst Stimulation (TBS) protocol (Li et al., 2019). Although the activity of the synapses can be regulated by interactions with interneurons and inhibitory neurons (e.g., Swanson and Maffei, 2019), the extensive and necessary response to an injury overwhelms any inhibitory or other influences that might be present.

The Glu released in response to the stimulation has two consequences. First, it creates both an electromagnetic (EM) field and a local field potential (LFP) that oscillate in synchrony with the synaptic activity in the space around the pyramidal neurons in 24P (Linden et al., 2010; Pockett, 2012; Hales and Pockett, 2014; Buzsáki et al., 2012; Ambron, 2023). Second, it induces a long-term potentiation (LTP) in the dendrites that is necessary for experiencing pain (Zhuo, 2014; Kang, 2015; Bliss et al., 2016; Lee et al., 2022). The LTP alters the transmission across the thalamo-pyramidal synapses so that even a few APs will elicit a response in the dendrites. This increased sensitivity, known as allodynia, explains why merely touching an injured area will cause intense pain hours or days after the injury occurred. The allodynia maintains an awareness of the injury site to avoid additional damage. Normally the pain disappears upon healing, but when it persists for 3 months or longer it is considered chronic and pathological. Since the duration of the allodynia is determined by the events associated with the LTP, characterizing these events is important for understanding persistent and chronic pain. We know a great deal about LTP from studies of learning and memory, both in invertebrates (Hawkins et al., 2006; Kandel, 2012) and in the mammalian hippocampus (Abel et al., 1997; Lømo et al., 2003; Bliss et al., 2016; Yu et al., 2022) and from the many studies from the Min Zhuo lab that are cited below. The electrophysiological events and biochemical reactions responsible for LTP are complex and are described in detail in a recent review (Lee et al., 2022). The following focuses only on the events that are essential to pain.

LTP is generally divided into an early phase that lasts hours and a late phase that lasts for days or even weeks. The early phase begins when the Glu released in response to inputs from the thalamus binds to α-amino-3-hydroxy-5-methyl-4-isoxazolepropionic acid (AMPA) receptors on the membrane of the post-synaptic dendritic terminals. The binding results in the entry of Na^+1^ and the generation of excitatory post synaptic potentials (EPSPs) in the pyramidal dendrites. The post-synaptic membrane also contains N-methyl-D-aspartate (NMDA) receptors that have a crucial function in regulating the transmission across the synapses in response to an injury or an inflammation, as well as in chronic pain (Blanke and Van Dongen, 2009; Li et al., 2019; Chen et al., 2021). The receptor is associated with a channel with a preference for Ca^+2^, but the channel is blocked by a tightly bound magnesium ion (Mg^+2^). When the Glu released by the APs generates a sufficient number of EPSPS, the block is removed and the influx of Ca^+2^ into the postsynaptic terminal initiates a series of enzymatic cascades in the early phase of LTP that are essential to experience pain (Zhao et al., 2005). Thus, the activation of the NMDA receptor directly links electro-physiological activity at the synapses to biochemical events in the dendrites.

The Ca^+2^ combines with calmodulin to form Ca^+2^/calmodulin (CAM), which activates CAM kinase IV and adenylyl cyclase-1 (AC-1). The development of LTP in the ACC of both mice and humans depends on the activation of AC-1 (Wei et al., 2002; Liauw et al., 2005; Liu et al., 2020; Shiers et al., 2022) and AC-1 activity is necessary for the expression of both visceral (Zhou et al., 2021) and neuropathic pain in mice (Vadakkan et al., 2006; Shiers et al., 2022). The effect is specific because deleting the AC-1 gene in the mouse ACC prevented the induction of LTP in the pyramidal neurons, but did not affect basal Glu transmission or acute pain (Wei et al., 2002; Li et al., 2019). In addition, injecting an AC-1 inhibitor directly into the mouse ACC alleviated chronic inflammatory pain without an effect on anxiety or fear (Kang et al., 2016). Since the ACC contains centers for mood and receives inputs from the amygdala for fear (Veinante et al., 2013; Figure 2), the finding that these behaviors were not affected by the inhibitor is another indication that AC-1 activity in 24P in the ACC is specifically associated with pain. Notable also is that studies of a mouse model of chronic inflammatory pain showed increased levels of AC-1 protein in the ACC that lasted for 2 weeks (Liu et al., 2020).

AC-1 synthesizes cyclic adenosine monophosphate (cAMP) that is required for the activation of Protein Kinase A (PKA). Activated PKA has a role in both phases of LTP. In the early phase, PKA contributes to the insertion of additional AMPA receptors into the dendritic membrane and it also phosphorylates the receptor making it more receptive to Glu (Chen et al., 2014; Song et al., 2017). Both events result in the initial sensitization of the synapse and the phosphorylation of the receptor is critical for nerve injury-induced behavioral sensitization. However, the early phase of LTP is transient because it depends on the phosphorylation of already existing proteins and can be reversed by phosphatases. In the late phase of LTP the PKA is transported to the cell body where it translocates into the nucleus and, together with CAM kinase, activates the cAMP Responsive Element-Binding protein (CREB; Toyoda, 2010; Kandel, 2012; Li et al., 2019). CREB is a transcription factor that binds to the cAMP Response Element (CRE) in the promoter regions of its target genes. CREB-mediated transcription results in the synthesis of proteins that alter the phenotype of the pyramidal neurons thereby maintaining the expression of LTP and the sensitization of the synapses (Toyoda, 2010; Kandel, 2012). Phenotypic changes are not readily reversed and the LTP can, in theory, last indefinitely. The binding of CREB to the CRE also results in the synthesis of proteins that contribute to synaptogenesis and neuronal plasticity (Redmond et al., 2002; Kandel, 2012; Ortega-Martínez, 2015). Taken together, these events will favor the circuits for pain over those less active and their persistence would contribute to chronic pain.

### Synaptic sensitization and oscillating electromagnetic waves

The data above establish that LTP, via the activation of the AMPA and NMDA receptors, AC-1 and PKA, is necessary for the expression of pain. Nevertheless, it is difficult to understand how the functions of these receptors and enzymes can result in the actual suffering of the pain because they are involved in the development of LTP in many other cortical neurons in response to various inputs and are even involved in learning and memory in invertebrates. Unless there is a consequence of these reactions that has yet to be discovered, there is no evidence that they are specific for pain.

The other consequence of activating the thalamo-dendritic synapses is the creation of the synchronized oscillating LFP and EM fields around the pyramidal neurons in 24P. The presence of both the fields and the pain can persist for a long time due to the NMDA-mediated changes in the phenotype of the pyramidal neurons. The question then becomes, do the LFP and EM fields have any role in creating painful experiences? The potential roles of LFPs in experiencing pain have been discussed previously (Ambron, 2023) and the focus here is on the EM waves.

### EM waves are associated with pain

The synchronized oscillating fields are the source of the EM waves that propagate through space (Pockett, 2012; McFadden, 2002; Buzsáki et al., 2012; Hales and Pockett, 2014). In addition to those from the ACC, pain-associated oscillating EM waves have been detected from other centers in the IPN, including the somato-sensory cortex, the thalamus, and both the IC and PFC. The frequency of the waves was not random, but fell into categories ranging from infralow <0.1 Hz to theta (4–7 Hz), alpha (8–13 Hz) beta (14–29 Hz) and gamma (30–100 Hz; Ploner et al., 2017). At present it is not possible to assign a specific function to any these waves, although there is some evidence that the waves from the ACC and IC in the high theta (6–9 Hz) and low beta (12–16 Hz) frequency ranges are important for chronic pain (Stern et al., 2005; Sarnthein et al., 2006). To begin to explore the potential role in pain of the waves emitted from 24P, we first need to examine the EM waves.

### The properties of EM waves

EM waves contain photons that travel at the speed of light in the vacuum of space. The photons contain information about their source that is encoded in their frequency, amplitude, and phase (MIT Technology Review, 2017; Müller et al., 2017) and since these parameters can take any value, there is a limitless number of unique waves. The oscillating EM waves emitted by large scale synchronized synaptic activity create the well-known brain waves that traverse the brain parenchyma to the scalp where they are detected by magneto-encephalopathy (MEG) or electro-encephalography (EEG). However, even a simple discrete movement of the hand generates an electromagnetic field from the motor cortex that can be detected in the opposite cerebral hemisphere (Brazdzionis et al., 2022). Moreover, the frequency and amplitude of the waves were reproducible, suggesting that it contained information that was a signature of the movement. This finding implied that EM waves can convey information between the two cerebral hemispheres, which is a departure from the traditional view that the interhemispheric exchange of information occurs via APs propagating along axons within the corpus callosum. Relevant therefore is the study by de Haan (2020) of so-called split-brain patients whose corpus callosum had been surgically severed to alleviate intractable epileptic seizures. When a noxious stimulus was applied to one hand, it was detected in both cerebral hemispheres by functional MRI. Thus, it appears that the EM waves that are emitted from the ACC and other pain centers can transfer information about pain from one hemisphere to the other.

EM waves contain energy and there are multiple lines of evidence that they can influence the activity of neuronal circuits as they traverse the brain (Richardson et al., 1984; Fröhlich, 2014; Ye et al., 2022). The influence was originally attributed to material particles, i.e., they had mass, but this is no longer correct. The influence is due to the photons and they cannot have mass because according to Einstein’s Special Theory of Relativity mass increases with speed and any material in the wave would become infinitely large at the speed of light, which is impossible. Instead, photons have an impact because they are packets (quanta) of energy. The following section explains how the photons in the EM waves from the 24P pyramidal neurons could alter the activity of target neurons.

### Transcranial magnetic stimulation

TMS introduces a transient electric current into a wire coil mounted on the scalp above a target area of the cerebral cortex (Klomjai et al., 2015). The current generates a magnetic field that alters the electrophysiological properties of the neuronal circuits in the cerebral cortex under the coil and therefore mimics the effects of endogenous EM waves. (e.g., Huerta et al., 2009; Alekseichuk et al., 2019). TMS can cause dramatic changes in behavior. Stimulating the cortex over Broca’s area, for example, interrupted speech mid-sentence, whereas TMS applied to the motor cortex elicited discrete motor movements (Cowey, 2005; Huerta et al., 2009; Klomjai et al., 2015). Repetitive TMS (rTMS) is a variation that applies pulses of electric current and its biological effects depend on the frequency intensity, and pattern of the stimulation protocol. Both TMS and rTMS are being used to treat depression (Peng et al., 2018) and other pathological brain conditions and they have had some success in alleviating pain (Chail et al., 2018; Fernandes et al., 2022; Kim et al., 2022). One notable outcome of rTMS is its ability to regulate LTP. In particular, theta-burst rTMS protocols, which evoked LTP in the pyramidal neurons in the ACC, influenced the initiation and maintenance of LTP in the hippocampus by altering the activity of AMPA and N-methyl-D-aspartate (NMDA) receptors (Vlachos et al., 2012; Komaki et al., 2014; Lenz et al., 2015; Rajan, 2017). Other frequencies activate GABAergic inhibitory neurons that depress the LTP. This means that the frequency of the EM waves emitted from the pyramidal neurons in the ACC after an injury could either induce or inhibit LTP in distant circuits.

## Discussion

### The functions of the EM waves: two theories

We can conclude from the studies cited above that the synchronized oscillating EM waves that emanate from the 24P pyramidal neurons after an injury are not a mere epiphenomenon or artifact of synaptic activity. Rather, the waves convey information about pain that can be distributed throughout the brain and they contain energy that can alter the activity of cortical circuits. Although the data clearly show that waves emitted from the ACC are important, the key question remains namely. How do the waves participate in experiencing pain? At present there is no way to answer this question. What follows is a theory in two parts that contains ideas that might be helpful and which can be tested. The first part concerns the potential role of the EM waves in the processing of sensory information. The second part explores the relationship between the waves, the brain and consciousness.

### The communication and integration of information

The pain that we ultimately experience from an injury depends on the exchange of information among the neuronal circuits in the thalamus, amygdala, NucAc, IC, and ACC, all of which are functionally and spatially distinct centers in the IPN. Less well defined, but equally important are inputs to the ACC from areas of the cortex that result in psychological pain. The role of the IC is essential because we can only focus on one sensation at a time (Qiu et al., 2014; Uddin et al., 2017; Bai et al., 2019). For example, pain is the predominate sensation after a lesion, yet it can be rapidly superseded by a distracting sensation, such as a loud sound or a brightly flashing light. It is even hierarchical, because the pain from a minor injury will be overwhelmed by that from a more serious injury. Likewise, attention to a red flower can be displaced by the sound of a siren or by the sudden remembrance of a psychologically painful experience (Weissman, 2005). This almost instantaneous shifting of attention allows us to adjust to rapid changes in our surroundings, but it requires a dynamic coordination and integration of sensory information at every instant in time. Explaining how all of this information is processed and assembled into a concept of the world is a version of the so-called combination problem originally posed by Chalmers (1995).

The neuronal centers in the IPN that are distributed throughout the brain communicate via the propagation of APs along axonal pathways within the network. Since APs propagate along myelinated axons at about 10 mm/ms, these electrophysiological processes are not rapid enough to explain the almost instantaneous processing of sensory information and focused attention, especially given the distances that must be covered. In their review on the role of EM waves in pain, Ploner et al. (2017) proposed that synchronized neuronal oscillations with distinct frequencies can serve the dynamic routing of information throughout the brain. If correct, this theory would have several significant implications: First, it would mean that EM waves distribute information among the widely separated centers in the IPN and that they do so orders of magnitude more rapidly than APs. Second, axons need not be involved, which is consistent with the findings by de Haan (2020) that EM waves transmit information between the cerebral hemispheres in the absence of the corpus callosum. Nevertheless, there must be some coordination between the waves and the APs (e.g., Vezoli et al., 2021) and how this occurs is an issue for future studies. And third, EM waves could rapidly integrate/collate the information that emerges from each of the centers in the IPN.

When EM waves with the same frequency interact, the amplitude of the emerging wave can be increased, decreased, or the waves can cancel each other, depending on their phase. Thus, if waves emitted from the amygdala are in phase with those from area 24P, their interaction would result in a wave with a greater amplitude. Since amplitude is a measure of the intensity of the energy in a wave, the emergent wave would have more energy that could result in a more intense pain. The waves for pain could also compete for attention with waves from other senses. The theta waves generated from the IC and ACC might be especially important in this regard. Studies of the hippocampus suggest that information contained within the theta rhythms from different sources interact to separate current sensory information from that retrieved from episodic memory (Kovács, 2020). The potential interactions among the waves is consistent with the theory proposed by Crick and Koch (1990) to explain the assembly of visual information, as well as theories by Varela et al. (2001), John (2002), Buzsáki (2006), and data from studies by Frohlich and McCormick (2013), Fröhlich (2014), and Vezoli et al. (2021).

What is important is that these ideas about the role of the EM waves in pain can be tested directly by using rTMS to deliver waves at designated frequencies, intensities, and phase into the cortex. The simplest experiment would be to generate waves with a frequency and phase that will cancel the waves that emerge from the ACC after an injury (see Pockett, 2012; Fröhlich, 2014; Ambron, 2023). In other words, block the waves block the pain. This is possible using available technology and there is a great advantage to this approach because it can be carried out in humans. There is already data indicating that rTMS can relieve pain (Paolucci et al., 2020). Moreover, if the frequency of the EM waves conveys specific information about pain, it could explain how burning pain differs from sharp pain and could provide insights as to the sources of the different types of psychological pain. These issues are difficult to approach in animal models, but are approachable in human subjects using MEG and rTMS.

### Where do we experience pain?

The proposals above still do not account for the suffering of pain. The EM waves emitted from the ACC contain information about pain that must somehow be transformed into an experience, but where and how this occurs is not known. There are several contemporary theories which propose that sensory information and experiences are linked to consciousness. According to the Integrated Information theory (IIT) by Tononi et al. (2016) and Tononi (2015), consciousness integrates the information from individual experiences that, in turn originate from each of our senses. The various iterations of the Global Workplace Theory posit that consciousness arises when sensory information is processed within a neuronal center (Baars, 1988; Baars et al., 2021). Interesting is that a component of this theory is a requirement for active NMDA receptors. Flohr (2006) has proposed that the return to consciousness after anesthesia depends on the activation of NMDA receptors. Both proposals are in agreement with the data showing that the activation of these receptors in the pyramidal dendrites is necessary to consciously experience pain. Important also is that the suffering associated with pain requires attention and the recent Network Synchronization (NetSync) theory connects experience and consciousness to circuits for focused attention, such as those in the IC (Nani et al., 2019). Finally, there are several theories based on empirical data that directly implicate EM waves in consciousness (Hunt, 2011; Pockett, 2012, 2013, 2017; Das, 2018; McFadden, 2020; McFadden, 2002; Young et al., 2022). Taken together, these theories suggest that the experience of pain is somehow connected to consciousness. But the nature of consciousness and where it resides are topics that have occupied philosophers for centuries.

Plato proposed that eternal, immaterial souls are separate from physical bodies and this idea was extended centuries later by René Descartes (1596-1650) who postulated that consciousness resides within the immaterial soul that is separate from the body, which is the domain of material things (Anglin, 2014). As the natural sciences progressed, the soul was equated with the mind and the body with the brain. The distinction between an immaterial mind and a material body is known as Cartesian dualism and it is but one theory about the nature of the mind (e.g., Smart, 2022). With regard to pain, all of the electrophysiological and biochemical data cited above supports the dualism concept. Thus, in the simplest case, the brain acquires information about an injury that is encoded in APs. The information in the APs is transformed into oscillating EM waves at the thalamo-pyramidal synapses in area 24P of the ACC. The waves, which now contain the information about the intensity of pain in their frequency and amplitude, enter the mind where the information is used to create a consciousness of pain. Note that this theory pertains only to the pain that arises directly from an injury or inflammation: it does not purport to explain how mood and psychological causes can influence pain. The theory, which is a synthesis of results obtained from cell and molecular biology and physics, does provide a framework to explore how our senses might contribute to a consciousness of the external world.

If each of our senses creates unique EM waves, then the information in each of these waves would, like those for pain, result in a sensory experience in the mind. The summation of all the experiences could then create a consciousness of the objects and events in the external world at every moment in time. This is a major departure from the traditional idea that all of the information that is needed to experience a sensation comes from activities in the brain. In other words, consciousness and other of our higher mental faculties are not due solely to the activities of the brain, but of the coordinated activity of both the brain and the mind. The theory also explains why, despite decades of trying, no center for consciousness has been found in the brain: it was not found because consciousness does not reside in the brain.

At present the mind is merely a location by default since we have no idea where the mind is located or how it works. However, given that the waves enter the portal to the mind, and that the waves can be studied and manipulated in humans, they should be useful tools to investigate the proposed relationships between the brain and the mind.

## Data availability statement

The raw data supporting the conclusions of this article will be made available by the author. Requests to access the datasets should be directed to richardambron5@gmail.com.

## Author contributions

RA: Conceptualization, Writing – original draft, Writing – review & editing.
